# On the Design of Broad-Based Neuropsychological Test Batteries to Assess the Cognitive Abilities of Individuals with Down Syndrome in the Context of Clinical Trials

**DOI:** 10.3390/brainsci8120205

**Published:** 2018-11-26

**Authors:** Ines A. Basten, Richard Boada, Hudson G. Taylor, Katherine Koenig, Veridiana L. Barrionuevo, Ana C. Brandão, Alberto C. S. Costa

**Affiliations:** 1 Division of Neurology and Epilepsy, Department of Pediatrics, Case Western Reserve University, Cleveland, OH 44106, USA; bastenines@gmail.com; 2 Department of Child and Adolescent Psychiatry, Universitair Ziekenhuis Brussel (UZ Brussel), B-1090 Brussels, Belgium; 3 Department of Pediatrics, University of Colorado School of Medicine, Aurora, CO 80045, USA; Richard.Boada@childrenscolorado.org; 4 Children’s Hospital Colorado, Aurora, CO 80045, USA; 5 Center for Biobehavioral Health, Nationwide Children’s Hospital Research Institute and The Ohio State University, Columbus, OH 43205, USA; hudson.taylor@nationwidechildrens.org; 6 Division of Developmental/Behavioral Pediatrics and Psychology, Department of Pediatrics, Case Western Reserve University, Cleveland, OH 44106, USA; 7 Department of Radiology, Cleveland Clinic Lerner College of Medicine, Cleveland, OH 44195, USA; koenigk@ccf.org; 8 Mellen Center, The Cleveland Clinic, Cleveland, OH 44195, USA; 9 Hospital Israelita Albert Einstein, São Paulo, SP, CEP 05652, Brazil; veridianaleiva@yahoo.com.br (V.L.B.); ana.cbrandao@einstein.br (A.C.B.); 10 Department of Psychiatry, Case Western Reserve University, Cleveland, OH 44106, USA

**Keywords:** down syndrome, hippocampal deficits, executive function, episodic memory, working memory, neurocognitive phenotype, memantine, NMDA receptor, Alzheimer’s disease

## Abstract

Down syndrome (DS) is the most common genetically-defined cause of intellectual disability. Neurodevelopmental deficits displayed by individuals with DS are generally global, however, disproportionate deficits in cognitive processes that depend heavily on the hippocampus and prefrontal cortex are also well documented. Additionally, DS is associated with relative strengths in visual processing and visuospatial short-term memory, and weaknesses in the verbal domain. Although reports of pharmacological rescuing of learning and memory deficits in mouse models of DS abound in the literature, proving the principle that cognitive ability of persons with DS can be boosted through pharmacological means is still an elusive goal. The design of customized batteries of neuropsychological efficacy outcome measures is essential for the successful implementation of clinical trials of potential cognitive enhancing strategies. Here, we review the neurocognitive phenotype of individuals with DS and major broad-based test batteries designed to quantify specific cognitive domains in these individuals, including the one used in a pilot trial of the drug memantine. The main goal is to illustrate the essential considerations in planning trials to enhance cognitive functions in individuals with DS, which should also have implications for the design of similar studies in individuals with other forms of intellectual disability.

## 1. Introduction

Down syndrome (DS) is the set of phenotypic features of variable expressivity that typically results from trisomy 21. It was first described by John Langdon Down in 1866 [1], and its genetic basis, an extra chromosome 21, was discovered almost 60 years ago by Jérôme Lejeune, Raymond Turpin, and Marthe Gautier [2]. DS occurs in 1 in 691 live births [3] and has a prevalence of 1 in 1000. The combined prevalence of DS in the United States and Brazil is estimated to be between 500,000 and 600,000 [3,4,5]. This number is expected to continue rising in both countries due to projected increases in the life expectancy of people with DS [5,6].

DS is the most common genetic cause of intellectual disability (ID) [3]. The brain phenotype for adults with DS is characterized by microcephaly, with disproportionally larger volume reductions seen in the hippocampus, prefrontal cortex, and cerebellum [7,8,9]. At birth, a wide range of studies of individuals with and without DS showed little or no appreciable anatomical differences [8,10]. However, several significant differences become apparent in the first few months of life, and include delayed demyelination, reduced growth of the frontal lobes, a narrowing of the superior temporal gyrus, diminished size of the brainstem and cerebellum, and a major reduction (20–50%) in the number of cortical granular neurons [7,8].

As individuals with DS age, they will inevitably develop a neuropathology indistinguishable from Alzheimer disease [9], which initially manifests itself in the mid-thirties to early forties [11,12]. This neurodegenerative process is thought to lead to the observed high prevalence of early-onset dementia in this population, most commonly occurring in the fifth or sixth decade of life [13,14]. The life expectancy of persons with DS is quickly approaching 60 years in the industrialized world and in many developing countries [13,15], mostly due to recent advances in the surgical and clinical management of the various comorbidities associated with DS [16]. Therefore, recognition and treatment of the developmental and neurodegenerative components of the syndrome may well constitute the two greatest unmet therapeutic needs of this population. Accordingly, there is an increasing demand for translational research aimed at effective treatment outcomes, which should ultimately lead to improved quality of life for individuals with DS and their families [17,18].

Our research team is particularly interested in the potential involvement of *N*-methyl-d-aspartate (NMDA) receptors and the therapeutic use of memantine in DS. Based on behavioral and electrophysiological data from mouse models of DS, we have hypothesized that NMDA receptor dysfunction may play significant pathogenic roles in both the neurodevelopmental and neurodegenerative components of DS [19]. These preclinical data led us to design a pilot clinical trial of memantine aiming to enhance the cognitive abilities of individuals with DS [20]. Due to its small sample size, this pilot study was expectedly inconclusive. However, *post hoc* power analysis of the resulting data was encouraging enough to warrant a Phase II, follow-up multicenter clinical trial of memantine in adolescents and young adults with DS [21].

The present paper critically reviews the current knowledge on cognitive deficits of individuals with DS and some of the broad-based neuropsychological test batteries used to assess cognitive skills in this population. We then describe the specific tests selected for the pilot memantine trial, their psychometric properties, and the rationale for administering these tests to evaluate the effects of memantine as a potential therapeutic agent in this population. The broader goal of the present work is to illustrate the essential considerations in planning trials to enhance cognitive functions in individuals with DS, which should also have implications for the design of similar studies for individuals with other forms of ID of known origin.

In summary, in addition to reviewing the literature on the neuropsychological assessment of individuals with DS, in the present work, we describe the psychometric properties of the test battery used in a pilot randomized, double-blind, placebo-controlled study of memantine in adults with DS. The data used here were derived retrospectively from deidentified, published material [20].

## 2. Neurocognitive Phenotype of Persons with DS

### 2.1. Intellectual Quotient

The best data available report the mean intellectual quotient (IQ) of school-aged children with DS to be in the low to mid 40s [22,23,24]. Carr [25] showed progressive reductions in IQ across the preschool period with IQs declining from a mean standard score of 70 at 6 months of age to a mean standard score of 50 by 4 years. These reductions are not due to an absence of learning, but correspond to an inability to keep pace with the cognitive development of same-age peers. Although the ID observed in persons with DS is indeed an across-the-board phenomenon, most studies examining the neurocognitive profile have shown disproportionate deficits in late-developing systems, including the medial temporal lobe (MTL), prefrontal cortex (PFC), and many of the neural systems that underlie language [8,26,27,28,29].

### 2.2. Hippocampus-Dependent Memory

The hippocampal complex is a neural structure that includes the hippocampus proper and the dentate gyrus [30]. It plays a central role in binding memories to distinct spatial and temporal contexts [31,32], which makes it critical for long-term memory (LTM) [33,34,35,36]. In humans, LTM can be classified as explicit (declarative) or implicit (non-declarative) memory. Explicit memory is recalled by a deliberate and conscious effort, such as factual knowledge of people, places and things. Implicit memory is reflected in unconscious manifestations of previous learning, as in faster responses to a previously cued category in making a perceptual judgment or in retention of a previously acquired procedure [37].

Individuals with DS perform especially poorly on tasks that fall into the category of explicit memory, which requires the recall or recognition of new information and is dependent on the functional integrity of the hippocampus [38,39]. In adults with DS, three studies analyzing various modalities of cognitive function demonstrated disproportional impairment in hippocampal function relative to global cognitive ability [40,41,42]. For example, Ellis et al. [42] compared individuals with DS to a typically developing (TD) group matched by chronological age (CA) on a task involving the visual LTM for pictures placed in different locations in a multi-page picture book. Consistent with hippocampal dysfunction, the DS group (mean age 26.8; range 14–51) was less able than the comparison group to recognize the pictures they had been shown or recall their locations.

In a study of adolescents, Carlesimo et al. [38] compared participants with DS to individuals with other forms of ID and to mental-age (MA)-matched TD children. The adolescents with DS performed less well on explicit memory tasks than both comparison groups. Compared to the ID group, individuals with DS performed particularly poorly in organizing verbal material according to its categorical structure and in actively retrieving stored information. The participants with DS displayed less efficient retrieval strategies as measured by a smaller discrepancy in recall of related vs. unrelated words, a reduced tendency to cluster words in recall, and higher scores on recognition relative to free recall trials. These results suggest weaknesses in both encoding and retrieval abilities as a potential basis for LTM deficits in persons with DS and are consistent with the structural MRI finding of reduced hippocampal volumes in a small sample of adolescents with DS [43].

Explicit memory deficits were confirmed by Pennington et al. [8]. In this study, a battery of 18 neuropsychological measures of prefrontal and hippocampal functions were administered to a sample of 20 school-aged individuals with DS (ages 11–19 years) and to 28 TD children (ages 3–6 years) individually matched on MA. The hippocampus-dependent measures used were the List Learning subtest of the NEPSY: A Developmental Neuropsychological Assessment, the Virtual Morris Water Maze Test, the Pattern Recognition Memory (PRM) and Paired Associates Learning (PAL) subtests of the Cambridge Neuropsychological Test Automated Battery (CANTAB), and the Ecological Memory Questionnaire. In spite of the large group difference in chronological age, the DS group performed worse than the MA group on all four of the hippocampal measures evaluated. Participants in the DS group tended to learn fewer words on NEPSY List Learning and spent significantly less time searching for the target object in the correct quadrant on the Morris Water Maze task in comparison with the MA controls. Compared to the MA-matched group, the participants with DS also had greater difficulty recognizing a previously presented pattern on CANTAB PRM and scored more poorly on CANTAB PAL. In contrast to the hippocampal measures, there were no significant group differences on individual measures of prefrontal function. On five of six measures, participants in the DS group tended to perform better than the MA-matched control participants. The authors interpreted the findings as providing evidence for a dissociation in DS between hippocampus-mediated LTM and prefrontal cortex-mediated working memory.

The timing of abnormalities in hippocampal development remains unclear [8]. Mangan [44] found deficits in place learning in toddlers with DS but not in the ability to make use of other types of cues as memory aides. The Place Learning Task required the use of cues in the surrounding environment to guide the search for hidden objects, whereas the other conditions allowed for searches that were driven by local landmarks and directional information in reference to the child’s own body. Conversely, Roberts et al. [45] suggested that DS-specific memory deficits are not yet evident in preschoolers with DS and emerge only gradually with age. The authors compared preschoolers with DS to TD children matched on receptive language or non-verbal scores as a proxy for mental age. Hippocampal function was assessed using a battery of eye-tracking and behavioral measures (object location retention memory, deferred imitation, A-not-B task, eye tracking task, and statistical learning). Findings failed to reveal significant group differences in either immediate or delayed memory on the eye-tracking or behavioral measures.

### 2.3. Executive Functioning

In addition to disproportionate deficits in hippocampal-dependent memory, there is also evidence for disproportionate prefrontal deficits in individuals with DS, although results have been mixed. Prefrontal functions, often grouped under the label of executive functions (EF), include the ability to hold information in the mind and manipulate it (i.e., working memory), inhibit actions for which a response tendency has been established (inhibitory control), and flexibly switch between response sets (set-shifting) [46].

Despite the lack of evidence from Pennington et al. [8] for disproportionate deficits in EF in individuals with DS, Lanfranchi et al. [47,48] documented impairments on dual-task measures of attention-switching in children and adolescents with DS compared to a TD group matched to the DS group on verbal ability. In a subsequent study, Lanfranchi et al. [49] compared 15 adolescents with DS to 15 TD children matched for MA on EF tasks assessing set shifting, planning/problem-solving, working memory, inhibition/perseveration and fluency, and sustained attention. The group with DS performed at a significantly lower level on tasks assessing set shifting, planning/problem-solving, working memory and inhibition/perseveration. The adolescents with DS completed the same number of pages on the sustained attention task as the TD children, but made a greater number of errors.

Rowe et al. [50] also found deficits on tasks assessing set-shifting, sustained attention and planning in comparing adults with DS, ages 23–40 years to controls with other forms of ID who were matched to these individuals on age and verbal ability. Similarly, Kogan et al. [51] found that adults with DS were impaired on visuospatial working memory tasks and on tests of visual-perceptual and visual-spatial reversal learning compared to individuals with Fragile X syndrome, but that the groups did not differ significantly on measures of spatial learning and object discrimination.

Studies of adults with DS also suggest that deficits in EF might be associated with the presence of dementia [49]. In a sample of 20 individuals with DS ages 22–58 years, Nelson et al. [52] found associations of age and dementia with lower scores on an object reversal learning task. Das et al. [53] compared a group of individuals with DS with controls without DS, matched for age and severity of ID, on a battery of tasks assessing planning and attention. Both groups were divided into ‘younger’ (40–49 years) and ‘older’ (50–62 years) subgroups. The authors found that the older DS subgroup performed more poorly on the measures than the younger DS subgroup. Because age was not associated with performance in the controls, the results were interpreted as evidence for early dementia in the older DS subgroup.

### 2.4. Short-Term Memory and Working Memory

Despite the ongoing debate about how best to distinguish between short-term memory (STM) and working (WM) memory, WM is generally regarded as a more active process in which mental representations are manipulated, whereas STM is viewed as a limited-capacity and more passive storage space [53]. These two types of memory are also considered to overlap, with STM serving as a component of WM. This conceptualization is consistent with the WM model of Baddeley and Hitch [54,55]. In this model, verbal-phonological (phonological loop) and visual-spatial (visuospatial sketchpad) representations (referred to respectively as the phonological loop and visuospatial sketchpad) are considered separate stores and are managed and manipulated with the help of attention-related processes termed collectively as the central executive [56].

Extensive research on verbal WM in individuals with DS reveals relative weaknesses on tests requiring repetition of digits or words in correct serial order. The number of digits TD children can remember in sequence increases from about three digits at the age 3 years to seven or eight digits at 16 years [57]. By comparison, persons with DS remember on average three or four digits [58]. Significant deficits in verbal WM in individuals with DS can still be found even in comparisons made with persons with ID of other etiologies matched by vocabulary knowledge [59,60,61,62,63] and non-verbal ability [64].

Although visuospatial STM or WM abilities appear to be better developed than verbal WM abilities, both types of skills are impaired relative to age-based normative standards [65]. Research also suggests that STM for visual-sequential information, as assessed for example by the Corsi Block Test, may be less impaired than memory for visual patterns [8,59,60,64,66,67,68].

### 2.5. Visuoconstructive Functions

Some spatial abilities are relative strengths for individuals with DS compared to mental age-matched groups [8]. For example, Silverstein et al. [69] found that individuals with DS (ages 3–56 years) performed better on drawing and other visuoconstructive tasks from the Stanford-Binet Intelligence Scale than individuals with ID matched on CA and MA who did not have DS.

### 2.6. Speech and Language

In contrast to relative strengths in visuospatial skills, individuals with DS have weaknesses in development of speech and language skills relative to MA expectations, especially in comprehension and use of the structural or morphosyntactical aspects of language [8]. Receptive and expressive vocabulary development and fast mapping, which is the speed of learning new vocabulary, are also impaired in relation to CA-matched controls, but are not compromised to the same extent as syntax [70,71]. Although receptive vocabulary is an area of relative strength, the depth of semantic processing may be more severely compromised in individuals with DS than anticipated based on their MA [72]. A study by Cleave et al. [73] examined narrative development across 1 year in children with DS aged 5–16 years. Across the year, the verbal narratives of children with DS developed in semantic complexity and global structure, with no growth in syntactic complexity or narrative length [70,73].

## 3. Neurocognitive Batteries to Assess Cognition in Individuals with DS

One of the challenges in evaluating the treatment effects of drug therapies is the selection of proper outcome measures to capture potential changes in clinical, cognitive and adaptive functioning in individuals with DS. Pharmacological trials in DS require measures that can be repeatedly and reliably administered across international sites to participants of varying ages and that are relatively unaffected by repeat administrations of the same of similar test items (i.e., “practice effects”) and sensitive to variations in skill in persons of both low and high general ability (i.e., devoid of “floor” or “ceiling” effects) [74]. Several studies have employed extensive test batteries to assess the neuropsychological profiles of individuals with DS. This section provides a brief review of some of the most prominent ones. 

### 3.1. The Study by Pennington and Colleagues

The study by Pennington et al. [8] can be seen as the prototype of large test batteries of measures designed to elucidate the relative strengths and weaknesses of individuals with DS. This study of school-aged children with DS and MA-matched TD controls employed the Scales of Independent Behavior-Revised (SIB-R) [75] to assess functional independence and adaptive functioning. A working group convened at the National Institutes of Health (NIH) [75] concluded that the SIB-R is an adequate tool for assessing potential improvements in adaptive functioning domains. It is suitable for a wide age range, has good psychometric properties, and has been used in previous intervention trials for DS [20,76]. The other components of the test battery developed by Pennington et al. are described below.

General intellectual ability was evaluated with the school-age version of the Differential Ability Scales (DAS). Although the entire DAS has not been used in clinical trials for DS [75], the individual subtests may be more appropriate for assessing cognitive strengths and weaknesses than test composites. The DAS is sensitive to variations in skills at the lower end of the ability range, is frequently used in studies with children with developmental delays, and has excellent reliability with an internal consistency score of 0.95 for the school-age level core [76]. Evidence for the validity of the DAS is provided by the high correlation of global scores with Full Scale IQ on the Wechsler Intelligence Scale for Children-Third edition (WISC-III) [8] (*r* = 0.85).

Language skills were assessed using the Test for Reception of Grammar (TROG), which evaluates receptive syntax skills. The TROG has an average internal consistency score of 0.77 across the ages of 4–9 years [77]. The TROG—second edition [78,79] is a promising measure but standard scores need to be expanded downward to accommodate lower performing individuals, and the measures need to be evaluated specifically for individuals with DS in terms of their psychometric properties [75].

The Word Structure subtest from the Clinical Evaluation of Language Fundamentals (CELF-3) was used to evaluate expressive syntax. Both the preschool [80] and the school-age [81] versions of the test were administered to avoid floor and ceiling effects. The CELF-3 Word Structure subtest has an internal consistency score ranging from 0.80 to 0.82 between the ages of 6 to 8 years, and the test-retest reliability is *r* = 0.76. Esbensen et al. [75] showed that in the DS population, expanded norms are needed for the CELF-3 that cover a broader age range and lower levels of functioning over the entire age range [8,75]. To evaluate verbal STM, participants completed the Recall of Digits subtest from the DAS, which is also a promising measure for clinical trials but needs to be further evaluated in individuals with DS [8,75].

The List Learning Test of the NEPSY (A Developmental Neuropsychological Assessment) was used to assess verbal learning and memory. This test has excellent reliability (*r* = 0.91; [82]) and imaging studies suggest the involvement of the posterior hippocampus in this type of supraspan learning task [83]. Further evidence for validity is provided by impairments in list-learning ability in patients with degeneration or damage to the hippocampus [8,84,85]. The List Learning Test is thus a promising tool for clinical trials in the DS population, but requires further investigation [75].

One of the tasks used to evaluate spatial LTM was a computer-generated Virtual Morris Water Maze Test [86], an adaptation of the Morris water maze task used as a rodent model of learning and memory [8]. The PRM and PAL tests from the CANTAB provided additional measures of long-term spatial memory. Prior research suggests that the PAL is a suitable task for individuals with DS and that it is sensitive to impairments in this population [20,74,75,87,88].

Several tasks of prefrontal function were also used. The CANTAB Stockings of Cambridge Test was administered to assess planning ability. Based on its similarity to the Tower of London (TOL) this task is assumed to reflect the integrity of the dorsal prefrontal cortex [8,89], although further research is needed to determine its utility in clinical trials for DS [75]. Verbal fluency was assessed using the NEPSY Verbal Fluency and Design Fluency tasks. NEPSY Verbal Fluency has a reliability of 0.74 for children aged 5 to 12 years and a Design Fluency task reliability of 0.59 in this same age range [8,90]. The NIH working group concluded that the NEPSY Verbal Fluency Test is an adequate test in individuals with DS [75]. The Stopping Task [91,92] was used to assess inhibition and CANTAB Spatial Working Memory (SWM) to assess spatial WM [93]. In the Stopping Task, the participant is required to press a button in response to a go signal, but on some trials is cued to inhibit this response. Inhibition is measured by the time needed to suppress the go response. CANTAB SWM requires participants to search under a series of colored boxes to locate a ‘‘blue token’’ hidden underneath one of the boxes. Although the psychometric properties of the stopping task for individuals with DS are largely unknown, SWM has been used in clinical trials and is an appropriate test in participants with DS [20,74,75,87,88]. Verbal WM was assessed using the Counting Span Task [94].

### 3.2. The Arizona Cognitive Test Battery (ACTB) for DS

Another test battery for individuals with DS, which was derived historically from the original work of Pennington et al. [8], was developed by Edgin and colleagues to assess prefrontal, hippocampal and cerebellar neuropsychological functions [88]. Given its origins, the ACTB is also based in part on tests from the CANTAB and it was designed to be sensitive to areas of specific cognitive impairments in individuals with DS [8,88,95,96]. To assess test-retest reliability (ICC) and practice effects, Edgin et al. [97] administered the ACTB to 54 youths with DS (ages 7–20 years) with a repeat administration of the battery 3 months after the initial assessment.

The ACTB includes the CANTAB PAL and the computer-generated Virtual Morris Water Maze Test from the Pennington et al. [8] test battery to assess hippocampal functioning. Although test-retest reliability was high for the PAL (ICC = 0.75), test-retest correlations for the Virtual Morris Water Maze Test indicated poor reliability (ICC = 0.43). Measures of prefrontal function include the CANTAB Intra-Extra Dimensional (IDED) Set Shift and Modified Dots tasks. IDED Set Shift task assesses set-shifting by requiring the participant to respond to different dimensions of a visual pattern in making a forced-choice discrimination. Previous research indicates differential impairment in patients with frontal lobe lesions, relative to lesions in patients with temporal lobe lesions or those with Alzheimer disease [98]. The Modified DOTS task [99] measures inhibitory control and WM and is suitable for participants aged 4 years to adulthood. The task requires the participant to press a button below a picture of a cat and to then shift to require pressing to a new location by the picture of a frog. The CANTAB IDED had low test-retest reliability (ICC = 0.48) as did the inhibitory control phase of Modified DOTS (ICC = 0.59). A further limitation of these and other tests of EF is their vulnerability to practice effects [75].

Tests of cerebellar function included in the ACTB are the CANTAB Simple Reaction Time (SRT) task, NEPSY Visuomotor Precision, and Finger Sequencing Task. The CANTAB SRT task measures simple reaction time. Participants press a button when a stimulus appears on a computer screen. Slowing of motor response time is typical with cerebellar dysfunction and studies have reported slowed reaction times in individuals with DS in comparison with MA controls and those with other developmental disabilities, such as autism [100]. This test has minimal language involvement and satisfactory test-retest reliability with no reported practice effects [101], suggesting that it would be suitable for clinical trials for DS. However, because this task would be difficult for persons with significant motor deficits, a simple reaction task requiring little to no fine-motor dexterity may have wider applicability [75,97]. The Finger Sequencing and the NEPSY Visuomotor Precision [82] and Finger Sequencing [102] also had good test-retest reliability [97] but are subject to this same limitation. Finger Sequencing requires the participant to generate sequences by tapping a number of fingers (1, 2, 3, 4) to a lever in succession. Visuomotor Precision is a timed measure hand-eye coordination that requires drawing of lines on paper within the borders of narrow tracks.

Several secondary measures are also part of the ACTB. Verbal comprehension and production were measured by the Kaufman Brief Intelligence Test, second edition (KBIT-II) verbal subscales [103], problem solving by KBIT-II Matrices, and immediate spatial memory by the CANTAB Spatial Span, a test modeled after the Corsi Block Task. The KBIT-II raw scores and CANTAB Spatial Span forward all demonstrated good to very good levels of reliability (ICC > 0.70 for all). Most Spatial Span measures had acceptable reliability, although reliability was low for errors (ICC = 0.42) and there was evidence that this test was more subject to floor effects than other CANTAB tasks. To decrease floor effects, CANTAB Spatial Span could be replaced by a table-top or more engaging version of the task such as actual Corsi blocks [104].

Caregiver ratings of participant behavior in the ACTB are the SIB-R [105] to evaluate adaptive behavior; Behavioral Rating Inventory of Executive Functioning (BRIEF) [106] to measure behavioral regulation and metacognition; and Nisonger Child Behavior Rating Form-Parent to assess conduct problems, hyperactivity, anxiety, sensitivity, ritualistic, stereotypic, social adaptive skill, and compliance [107]. Although most of these measures were stable across the follow-up period (ICCs > 0.8), some of the individual scales, such as BRIEF Working Memory T-score and Nisonger Self-Injury/Stereotypic, had poor reliability or were subject to large practice effects. Parent reports can provide useful information as secondary measures and may enhance ecological validity because of their relevance to daily life functioning and well-being. However, these measures are also susceptible to placebo effects [108] and thus are unlikely to be useful as quantitative measures of primary outcome in clinical trials.

### 3.3. The Test Battery by Liogier d’Ardhuy and Colleagues

In a 6-month longitudinal and multinational study of cognitive function in individuals with DS, Liogier d’Ardhuy et al. [74] used subtests of the Repeatable Battery for the Assessment of Neuropsychological Status (RBANS) to assess immediate memory (List Learning) and language capacities (Picture Naming and Semantic Fluency) [109]. Findings indicating that List Learning had good test-retest reliability (ICC 0.69 for adolescents and ICC 0.64 for adults), are not subject to floor effects, and are sensitive to variations in age and IQ, suggesting that it is suitable for clinical trials for DS [75]. Semantic Fluency was also reliable (ICC adolescents 0.59; ICC adults 0.73), with no evidence of floor effects, while Picture Naming had low test-retest reliability (ICC < 0.53 in both groups). Although the Story Memory Test from this battery had moderate reliability (ICC 0.69 for adolescents and ICC 0.67 for adults), it was subject to floor effects. Practice effects were also evident in gains in scores in adolescents with DS over the 6-month follow-up period.

WM was assessed by Liogier d’Ardhuy and colleagues using the CANTAB Spatial Span (SSP), a measure that has also been used in clinical trials for attention deficit hyperactivity disorder [110]. Participants with DS performed poorly on both the digits forward and backwards components of the test, with many unable to recall any digits in reverse order. The ICC for the forward digits portion of SSP was 0.67 for adolescents and 0.55 for adults and is considered a good measure of frontal function [75]. Reliability for the backward portion was low with evidence for floor effects.

Liogier d’Ardhuy et al. used the Clinical Evaluation of Language Fundamentals-Preschool-2 (CELF-P2) [111] to assess language. Normed for preschool-aged children 3-6 years, the CELF-P2 comprises tests of different aspects of language. Word Classes evaluates the participant’s ability to understand and express relationships between semantically related words. This measure is stable, reliable, and sensitive to age and IQ variations in individuals with DS, although it is subject to ceiling effects in adults.

These authors had caregivers complete the preschool version of the BRIEF (BRIEF-P) to assess behavioral correlates of executive dysfunction. The BRIEF-P yields a summary measure of executive dysfunction, the Global Executive Composite (GEC), as well as scales for specific types of deficits in behavioral self-regulation and organizational skills [106]. In one study, the BRIEF-P yielded a unique pattern of strengths and weaknesses in young children with DS, showing impairments in WM and planning but not in inhibition or emotional control [96]. The BRIEF-P is reliable, stable and sensitive to age differences. This measure can be used to detect impairment in the WM domain and is suitable for adolescents with DS aged 12–17 years.

The Leiter International Performance Scale Revised (Leiter-R) [112] was used by Liogier d’Ardhuy et al. to explore the influence of age variations (adolescents vs. adults) on non-verbal IQ level, although findings for the original version suggested a floor effect. However, scores on the more recent version of this test, the Leiter-3, were well distributed without evidence for a floor effect and thus may be more suitable for measuring variations in ability at the lower end of the IQ range [74,75].

### 3.4. The TESDAD Study Group’s Test Battery

De Sola et al. [87] administered a test battery referred to as TESDAD to 86 young adults (ages 16-34 years) with DS and an age-matched control group of normally developing adults. Similar to the ACTB, this battery consisted of several tests from the CANTAB [113] along with standardized paper and pencil tests. CANTAB Motor Screening was administered to assess psychomotor speed. Measures of EF included the SRT and SSP from the CANTAB, and Digit Span Forward and Backward from the Wechsler Adult Intelligence Scales, 3rd Edition (WAIS-III) to assess attention span, STM, and WM; a word generation task requiring production of animal names to assess semantic fluency; the Drexel University version of the Tower of London (child’s version) to assess planning; Weigl Color-Form Sort Test to evaluate mental flexibility; and Cats and Dogs Test to measure response inhibition. Measures of visual episodic memory and learning were obtained using the CANTAB PAL and PRM. Verbal episodic memory was assessed using the Cued Recall Test (CRT), a test requiring participants to recall verbal information (98). Most of these measures are regarded as appropriate tests to use in assessing individuals with DS, although CANTAB PRM and the Weigl Color-Form Sort Test are considered promising but in need of further study [75]. In addition, other investigators have shown test-retest reliability to be low for CANTAB SSP backward recall [74]. Measures of expressive and receptive language in the TESDAD included the Boston Naming Test and Token Test. The Boston Naming Test has proven to be an appropriate task for individuals with DS [75]. Specific test-retest analysis and evaluation of potential practice effects for the TESDAD are not yet available.

## 4. Pilot Clinical Trial of Memantine in Young Adults with DS

Preclinical evidence of the efficacy of memantine in the Ts65Dn mouse model of DS led to the design of a pilot, phase IIa clinical trial to investigate if these findings have therapeutic implications for individuals with DS [20]. Memantine is a drug approved by the United States’ Food and Drug Administration (FDA), the European Medicines Agency Europe, Brazil’s National Health Surveillance Agency (ANVISA), and federal agencies in several other markets for the treatment of moderate-to-severe dementia caused by Alzheimer disease [19]. In spite of its small scale, the memantine pilot trial was historically relevant as the first clinical study in DS to benefit fully from the lessons learned from both preclinical work in animal models and contemporary neuropsychological research on this population.

The Ts65Dn mouse is still the best-studied and the most complete mouse model for DS in terms of displaying phenotypes mimicking what is observed in persons with DS [114]. Studies on these mice suggest that learning and memory deficits on tests dependent on the functional integrity of the hippocampus may be attributable, at least in part, to altered signaling via *N*-methyl-d-aspartate (NMDA) receptors [115]. Furthermore, pharmacological experiments with the uncompetitive, moderate-affinity *N*-methyl-d-aspartate (NMDA) receptor antagonist, memantine, have produced rescued performance in behavioral tests of learning and memory in Ts65Dn mice [116,117,118]. Additionally, alterations in two types of synaptic plasticity in the hippocampus, NMDA receptor-dependent long-term depression (LTD) and theta-burst stimulation-induced long-term potentiation (LTP), can be reset to levels comparable to those observed in euploid control mice with the use of therapeutically relevant doses of memantine [119,120].

The pilot memantine clinical trial failed to reveal significant differences between the memantine and placebo groups on the two primary outcome measures. However, significant performance improvements were seen in the memantine group on the California Verbal Learning Test-II (CVLT-II) Short Form (*p* = 0.046) compared with the placebo group. The CVLT-II measures supraspan word learning ability as an index of episodic verbal LTM. Similar to the List Learning Test of the NEPSY, scores on this test are known to reflect posterior hippocampal functioning (also based on neuroimaging) and are impaired in patients with various forms of degeneration or damage to the hippocampus [20]. Additionally, group differences on one of the primary outcome measure scores, the number of stages completed on the PAL (a measure requiring learning of the locations of abstract visual patterns), approached significance, with a *p*-value of <0.10. The Recall of Digits Forward Test from the Differential Ability Scales, 2nd Edition (DAS-II) also approached significance [20]. In this task, the participant was asked to repeat, in the same order, an increasingly longer string of single-digit numbers verbally read aloud by the examiner.

Equally important for the objectives of the trial, the use of memantine was well tolerated, with only infrequent and mild adverse events noted. According to caregivers, two participants showed increased anxiety, one complained of dizziness for a few days, and one displayed increased self-talk. Thirty-seven of the 40 enrolled participants completed the trial. This trial was one of very few placebo-controlled trials to be performed in individuals with DS and was a necessary step toward the establishment of a bridge between preclinical work with animal models and the investigation of a fuller range of pharmacotherapies to improve the quality of life of individuals with DS [20].

### 4.1. Selection of the Test Battery for the Pilot Memantine Trial

The neuropsychological tests used for the memantine pilot trial were selected in consultation with Professor Bruce Pennington. Therefore, this test battery shared many components found in the original work led by him. The primary efficacy measures of this trial consisted of potential improvement in CANTAB PAL and PRM test scores from the baseline session to the second testing session at 16 weeks of memantine treatment. Two secondary measures of hippocampus-dependent function were also administered: the CVLT-II Short Form [121] and the Rivermead Behavioral Memory Test-Children’s version (RBMT) [20]. Although the battery had a similar composition to what had been used in other research with individuals with DS, such as the ACTB [88], it was designed to be comprehensive while avoiding undue burden on the trial participants.

There are some aspects of this test battery that are important to highlight. First, the measures were selected in order to minimize floor effects in participants with DS. Therefore, approximate mental age was used to gauge the appropriateness of a measure, and existence of chronological age norms was only a secondary consideration. Given that the battery was designed to be used longitudinally in a clinical trial, participants acted as their own controls, and comparison of raw scores over time was the critical comparison.

Second, some few measures were selected primarily to characterize the sample. These are the PPVT, Matrices of the DAS-II, and SIB-R. The PPVT and SIB-R have norms that span a wide age range, and include the chronological age range of our targeted participants. The Matrices subtest of the DAS-II does not go into adulthood, but uses a Rasch modeling approach that selects an appropriate item set for each participant based on his or her ability level, in order to prevent floor or ceiling effects. Ability scores can be compared, and mental ages can also be derived.

Third, the battery was devised to include measures of cognitive domains (i.e., discriminant measures) that were not hypothesized to be affected by the drug memantine. Based on prior medical literature as well as animal work with the DS mouse model, the mechanism of action of memantine was hypothesized to affect temporal lobe-dependent memory primarily. Thus, having measures of other domains, such as language, would allow us to detect whether memantine had a specific effect on targeted cognitive domains, rather than affecting all domains. If the latter were the case, alternative mechanisms would have to be explored.

Fourth, STM, episodic memory, and executive function domains were measured using at least two measures each, in order to be able to capture some of the heterogeneity of these domains. In the STM area, we included both verbal and non-verbal tasks. For episodic memory, our primary outcome domain in the clinical trial, two scores from the CVLT-II, were used, one capturing total items correctly recalled during learning trials, and the other taking into account false positives (i.e., intrusions). In the visual domain, a lower level pattern recognition task was used, as well as a higher order task that required pairing non-namable stimuli to a specific location. This approach to battery selection allows flexibility in creating composite scores; one can compute them by domain (e.g., STM vs. episodic memory) or by modality of presentation (e.g., verbal vs. visual).

Lastly, the contents of the battery were selected with pragmatic considerations in mind. It is mostly visual, which is an easier modality to administer to participants with DS. Computer-based tasks (such as the CANTAB) are generally colorful, interesting and interactive, which helps keep participants motivated and engaged. Half of the verbal tasks only required motor responses (e.g., pointing), which minimized confounds introduced by common dysarthric, apraxic and articulatory deficits. Even the two tasks that required verbal production had a constrained set of responses. The target items of the CVLT-II and the digits to be repeated during Recall of Digits are known to the examiner, thus making it easier to code responses. Only intrusion errors were open ended, and our administration protocol has a parent or caregiver write down responses alongside the examiner, maximizing the probability of correctly identifying the participant’s verbalization.

### 4.2. Test-Retest Reliabilities for Measures Used in the Pilot Memantine Trial

In cognitive studies, an inter-rater reliability coefficient greater than 0.9 is considered excellent, a coefficient between 0.8 and 0.9 is considered good, and a coefficient between 0.7 and 0.8 represents adequate reliability. Here, we performed a test-retest reliability (Pearson r) analysis on the data obtained from 19 participants in the placebo arm of the pilot memantine study. Results of this analysis are summarized in Table 1. As can be seen in this table, four of the measures used had excellent reliability (SIB-R, NEPSY Verbal Fluency, PPVT-III receptive vocabulary, and CANTAB SWM Between errors score). The TROG-2, measuring comprehension of grammar and syntax, and the CANTAB PAL Stages Completed (one of the scores for episodic visual memory) had good reliability. Most other measures had adequate test-retest reliability (i.e., approximating, or above 0.7), whereas only one of the indices from the CANTAB SWM Test (i.e., Strategy score) had poor test-retest reliability (*r* = 0.33).

It is interesting to note, that if one looks at tests for which various sub-measures were generated, different indices may produce similar or different levels of reliability. For example, the two scores for the CVLT-II displayed adequate test-retest reliability. In contrast, the CANTAB SWM has one score that had excellent reliability (between errors), while the strategy score was poor. One way of interpreting this discrepancy in the latter subtest is that it is primarily measuring a simpler aspect of WM (i.e., closer to spatial span). In other words, participants may keep track of where they have just looked for a chip, and where the computer may have hidden previous chips, but they are not consistent in applying higher order strategies. Therefore, the strategy score is probably not very meaningful for this population.

Also interesting was the finding that the Matrix Reasoning Test from the DAS-II had lower test-retest reliability than expected, especially given its non-verbal nature. Other batteries used with participants with DS have attempted to minimize the verbal loading of tests, but in fact, all the tests in our battery that had verbal loading or measured verbal skills directly had higher test-retest reliabilities than the Matrices Test (i.e., PPVT-III, NEPSY Verbal Fluency, TROG-2, CVLT-II, Recall of Digits).

Measures that had a verbal loading/verbal content can be divided into those requiring a verbal response (CVLT-II; Recall of Digits) and those that just required a motor (pointing) response (PPVT-III, TROG-II). As expected, reliability coefficients for the latter were slightly higher than for the former, but even the ones for which a verbal response was necessary had adequate reliability. One caveat, however, is that the examiners in the pilot memantine study were highly trained neuropsychologists who were experienced in testing patients with verbal and articulatory deficits.

Lastly, it was useful to learn that parent/caregiver ratings of the adaptive functioning of participants with DS using the SIB-R were highly reliable across a 16-week interval. It should be noted that the broad independence standard score captures adaptive skills across domains. A reliability coefficient for the maladaptive scores of the SIB-R cannot be computed as the scores are not normally distributed.

### 4.3. Follow-up Memantine Trial in Adolescents and Young Adults with DS

Because of the promising findings from the pilot study, and memantine’s positive safety profile [20], the research team planned and initiated a larger follow-up trial. One set of *Post hoc* power analyses of the results obtained in the pilot study showed the requirement of a minimum sample size of 48 participants per group to demonstrate a significant difference in CVLT-II Short Form scores between the medication and placebo arms of the study with 80% power and a two-tailed hypothesis. For the Recall of Digits Forward test, this number increases to 55 participants per group. For the number of stages completed on the PAL, the target number would be 79 participants per therapeutic arm. The specific calculation results of required sample sizes that we are presenting here for the follow-up memantine study were performed using the sample size calculator from the ai-therapy.com website [122] using the effect sizes (Cohen’s d) from the neuropsychological variables assessed in the pilot memantine trial [20]. (Other *Post hoc* power analysis methods were also used with similar results.) In accordance with these calculations, we determined that in the follow-up, confirmatory study, we would be recruiting a total of 200 individuals with DS (i.e., 100 participants in the memantine arm and 100 participants in the placebo arm). This new trial [21] is a prospective, double-blind, placebo-controlled, randomized 16-week test, which follows a protocol modeled after the one used in the pilot trial.

The larger sample size is currently being recruited at two sites (University Hospitals Cleveland Medical Center, Ohio, USA and Albert Einstein Israelite Hospital, São Paulo, Brazil). As with the pilot study, the drug dosage follows the standard titration of memantine for the treatment of Alzheimer disease. In this trial, we have expanded the age range of the participants from 18–32 years to 15–32 years. The test battery for the ongoing follow-up memantine trial includes measures assessing skills in five domains: memory, intellectual functioning, language and vocabulary, visual and verbal WM, and adaptive/behavioral functioning. Some measures were selected based on their sensitivity to the types of changes anticipated from the putative mechanisms of action of the drug, whereas some were simply selected based on results from the pilot trial [20]. We hypothesize that the participants in the memantine arm of the trial will show a greater improvement from baseline to the 16-month visit than the placebo group on measures of declarative episodic memory, and that this improvement may also be evident on measures of WM. Because of the trend toward significance for the Recall of Digits test, this test will also be administered in the new trial, along with two additional prefrontal tasks: CANTAB SSP and a Go/No-go test. We have also selected measures of receptive semantics and grammatical understanding that we predict will remain relatively stable, thus acting as benchmarks against which to compare the anticipated improvements in memory and WM. A secondary hypothesis is that memantine may decrease the frequency or severity of behavioral difficulties, although we found no indication of this potential in the previously mentioned pilot trial. To test this hypothesis, the SIB-R will also be administered. We plan to discuss the design of this follow-up study in a separate paper.

## 5. Discussion

The average IQ of school-aged children with DS is in the low to mid 40s [22,23,24], which clearly means that the cognitive deficits associated with this genetic disorder are global in nature. However, findings from multiple studies challenge the view that these individuals have similar impairments across cognitive domains [26] and suggest disproportionate deficits in hippocampal and prefrontal cortex-dependent functions in the context of this global cognitive deficit [8,22,104]. In the present report, we described the range of neurodevelopmental deficits associated with DS and previous neuropsychological test batteries that have been developed for this population.

Research on potential therapies aimed at addressing the neurodevelopmental and neurodegenerative components of DS are beginning to benefit from insights gained through translational studies in the Ts65Dn and other mouse models of DS. For example, findings stemming from studies of the Ts65Dn mouse, together with the FDA approval of memantine as a treatment for Alzheimer disease, raised the possibility that this drug could be of benefit to persons with DS and paved the way for a pilot clinical trial to determine if similar effects would be observed in individuals with this genetic disorder. In that study, neuropsychological assessment was performed at the beginning of the study and after the 16-week trial of either memantine or placebo. The test battery was based on the one used by Pennington et al. [8], and examined a wide range of neuropsychological abilities. Although the benefits of memantine were much less impressive that those found in Ts65Dn mice, improvement in a supraspan measure of word learning assessing episodic verbal LTM was significantly greater for individuals treated with memantine. In addition, two other measures approached significance and the treatment was well tolerated. This pilot study was one of the very few placebo-controlled trials to be performed in individuals with DS, and was a necessary bridge between the preclinical work on animal models of DS and more intensive investigations of pharmacotherapies to improve the quality of life in individuals with DS [20].

As is generally the case for small-scale clinical studies, the results of the memantine pilot study were used to inform the design of a larger trial. The detailed description of the test battery of neuropsychological assessments being used in this phase II, follow-up memantine clinical trial is beyond the scope of the present review and will be discussed in a future paper.

We would like to acknowledge that latent trait modeling and confirmatory factor analyses, instead of the calculation of a simple Pearson r, would have been more reflective of the current methodological best practices related to understanding the reliability of neuropsychological measures in the pilot memantine trial. However, it is almost impossible to do this type of analysis with the small sample size from the pilot study, which had 40 participants in total and only 20 participants in the placebo arm. In contrast, latent trait modeling typically requires sample sizes of 100 or more for the models to have a good chance of converging and producing convincing fit statistics. Accordingly, we definitely plan to attempt to perform these more sophisticated analyses on the data to be derived from our Phase II, follow-up memantine study, given that we should have a large enough sample when that study is concluded.

It is easy to argue that harmonization of test batteries across studies would be highly desirable, so that at least baseline results could be compared and aggregated across sites performing trials of different pharmacological agents. Although this is undoubtedly true in present international efforts to better understand the neurodevelopmental profile of individuals with DS in general, these are early days in the area of pharmacological interventions aimed at enhancing cognitive skills in these individuals. Much is still needed to be learned in terms of what is the minimum set of informative measures that needs to be included in each trial, and it is equally important not to stifle innovation in this emerging field of inquiry. Present attempts to create standardized comprehensive test batteries are at a very preliminary stage. The few batteries that have been developed, including the ones we have used in our own clinical trials, are variations of those employed in the descriptive studies that were reviewed here. This previous research has guided the selection of our test battery by providing information on the psychometric properties of our measures and has reinforced the need to examine the potential benefits of a clinical intervention on multiple cognitive domains.

In the same year the pilot memantine trial was published, Hanney et al. [123] published another clinical trial with memantine in adults with DS 40 years of age or older. This was a randomized, double-blind, placebo-controlled trial to assess the safety and efficacy of memantine in improving cognitive and adaptive function in older individuals with DS. The primary endpoints were changes in cognitive and adaptive functioning as measured by the Down Syndrome Attention, Memory and Executive Function Scales (DAMES) and the Adaptive Behavior Scale (ABAS) parts I and II. The authors found that the treatment with memantine was well tolerated in their participant sample, but that the treatment produced no significant improvement on neither the primary nor the secondary efficacy measures. A likely explanation for the lack of efficacy in that well-designed trial (as well as in many Alzheimer disease trials) is that irreversible neurodegenerative cascades were already well underway to the point that functioning could no longer be restored by the time pharmacological treatment was attempted [20]. Still, more recent work by this same research team has shown that anti-dementia drug treatment with relevant drugs (donepezil, galantamine, rivastigamine, and memantine) delivered a significant survival advantage to individuals with DS and dementia compared to those who were not prescribed these medications [14]. This provides evidence that even in older individuals with DS, anti-dementia drug treatment may not be futile.

It will be important to consider conducting trials on younger children with DS. Treatment of DS-specific memory deficits, for example, may be most effective if administered earlier in life as a means for preventing or reducing progressive worsening of these problems with age [45]. Early attempts to expand trials to younger participants are illustrated by studies by Spiridigliozzi and colleagues [124] and Kishnani and colleagues [125], who examined the effects of rivastigmine and donepezil, respectively, to improve cognitive function in school-aged children with DS. Although these studies failed to demonstrate improvements in performance on the selected measures, their methodologies were innovative and merit consideration in future trials of drug and other therapeutic strategies to enhance cognitive function and quality of life in individuals with DS and their families. Clinical trials targeting functional change over extensive follow-up periods will also be needed, as positive effects of interventions may require several years to fully manifest [20].

Finally, irrespective of recent or future advancements in the area of pharmacological therapeutics, it is quite clear that developmental interventions involving speech, physical and occupational therapies, as well as special educational programs, will likely continue to be the mainstay approaches to improve cognition and adaptive skills in young children with DS for the foreseeable future [126,127]. However, it is also important to note that evaluating intervention studies in these areas faces the same challenges encountered in the study of potential pharmacological interventions, and that efficacy is often not assessed as rigorously as one would like in the field of habilitative interventions in DS.

## 6. Conclusions

In the present study, we have critically reviewed the current knowledge on cognitive deficits of individuals with DS and some of the broad-based neuropsychological test batteries that have been used to assess cognitive skills in this population. We also described the specific tests selected for a pilot trial of the drug memantine on enhancing the cognitive skills of young adults with DS, including brief descriptions of the psychometric properties of each measure and the rationale for administering such tests in the context of clinical trials in this population. The broader goal of the present work was to illustrate essential considerations in planning trials to enhance cognitive functions in individuals with DS, such as a follow-up phase II trial of the drug memantine currently underway. The field of pharmacological enhancement of cognitive abilities of persons with DS is still in its infancy, with the basic principle that such interventions are even possible still awaiting to be strongly proven. In examining several broad-based neuropsychological test batteries, some basic agreement emerges in terms of the choice of a few computer-based tests (e.g., CANTAB PAL and PRM). However, much remains to be learned in terms of what is the minimum set of informative measures that should be included in each trial, and how much customization, based on for example knowledge of mechanisms of drug action, will be necessary for each study. Therefore, one cannot overemphasize how critical it is for the few active groups in this area to remain humble. We should acknowledge how little we still know in this early stage of our shared journey of designing pharmacological interventions designed to enhance the quality of life of those with DS and their families, which is perhaps the best argument against any premature attempts to stifling innovation in this fledgling field. We should also concede that there is no shortage of knowledgeable and well-intentioned professionals who still believe that each of such attempts is no more than a fool’s errand. Not to mention those who, for various historical reasons, would go out of their way to prevent the implementation or try to abort new studies in this area.

## Figures and Tables

**Table 1:** Pearson *r* values for test-retest reliability for various measures used in the pilot memantine trial.

Measure	Test-Retest Reliability (Pearson r)
CVLT-II—Total Recall Discriminability	0.75
CVLT-II—Total Recall Score	0.76
CANTAB PRM—Raw Score Correct	0.68
CANTAB SWM—Between Errors	0.93
CANTAB SWM—Strategy Score	0.33
CANTAB PAL First Trial Memory Score	0.72
CANTAB PAL Stages Completed	0.81
Rivermead Total Score	0.62
DAS-II Recall of Digits Ability Score	0.74
DAS-II Matrices Ability Score	0.62
TROG-II Item Correct Raw Score	0.81
PPVT-III Standard Score	0.93
NEPSY Verbal Fluency Total Raw Score	0.92
SIB-R Total Independence Standard Score	0.95

## References

[1] Down J.L.H. (1866). Observations on an ethnic classification of idiots. Lond. Hosp. Clin. Lect. Rep..

[2] Lejeune J., Turpin R., Gautier M. (1959). Le mongolisme premier exemple daberration autosomique humaine. Ann. Genet.-Paris.

[3] Parker S.E., Mai C.T., Canfield M.A., Rickard R., Wang Y., Meyer R.E., Anderson P., Mason C.A., Collins J.S., Kirby R.S. (2010). Updated national birth prevalence estimates for selected birth defects in the united states, 2004-2006. Birth Defects Res. A Clin. Mol. Teratol..

[4] Brandão I.M., Fonseca V., Madi R.R. (2012). Prevalence of People with down Syndrome in Brazil. www.scientiaplena.org.br.

[5] De Graaf G., Buckley F., Skotko B.G. (2017). Estimation of the number of people with down syndrome in the united states. Genet. Med..

[6] Bittles A.H., Glasson E.J. (2004). Clinical, social, and ethical implications of changing life expectancy in down syndrome. Dev. Med. Child Neurol..

[7] Nadel L., Tager-Flusberg H. (1999). Down syndrome in cognitive neuroscience perspective. Neurodevelopmental Disorders.

[8] Pennington B.F., Moon J., Edgin J., Stedron J., Nadel L. (2003). The neuropsychology of down syndrome: Evidence for hippocampal dysfunction. Child Dev..

[9] Lott I.T., Dierssen M. (2010). Cognitive deficits and associated neurological complications in individuals with down’s syndrome. Lancet Neurol..

[10] Schmidt-Sidor B., Wisniewski K.E., Shepard T.H., Sersen E.A. (1990). Brain growth in down syndrome subjects 15 to 22 weeks of gestational age and birth to 60 months. Clin. Neuropathol..

[11] Leverenz J.B., Raskind M.A. (1998). Early amyloid deposition in the medial temporal lobe of young down syndrome patients: A regional quantitative analysis. Exp. Neurol..

[12] Zigman W., Schupf N., Haveman M., Silverman W. (1997). The epidemiology of alzheimer disease in intellectual disability: Results and recommendations from an international conference. J. Intellect. Disabil. Res..

[13] Zigman W.B., Lott I.T. (2007). Alzheimer’s disease in down syndrome: Neurobiology and risk. Ment. Retard. Dev. Disabil. Res. Rev..

[14] Sinai A., Mokrysz C., Bernal J., Bohnen I., Bonell S., Courtenay K., Dodd K., Gazizova D., Hassiotis A., Hillier R. (2018). Predictors of age of diagnosis and survival of alzheimer’s disease in down syndrome. J. Alzheimers Dis..

[15] Patterson D., Costa A.C. (2005). Down syndrome and genetics—A case of linked histories. Nat. Rev. Genet..

[16] Roizen N.J., Patterson D. (2003). Down’s syndrome. Lancet.

[17] Gardiner K.J. (2010). Molecular basis of pharmacotherapies for cognition in down syndrome. Trends Pharmacol. Sci..

[18] McCabe L.L., Hickey F., McCabe E.R. (2011). Down syndrome: Addressing the gaps. J. Pediatr..

[19] Costa A.C. (2014). The glutamatergic hypothesis for down syndrome: The potential use of n-methyl-d-aspartate receptor antagonists to enhance cognition and decelerate neurodegeneration. CNS Neurol. Disord. Drug Targets.

[20] Boada R., Hutaff-Lee C., Schrader A., Weitzenkamp D., Benke T.A., Goldson E.J., Costa A.C. (2012). Antagonism of nmda receptors as a potential treatment for down syndrome: A pilot randomized controlled trial. Transl. Psychiatry.

[21] NCT02304302. NCT02304302.

[22] Carr J. (1988). Six weeks to twenty-one years old: A longitudinal study of children with down’s syndrome and their families. Third jack tizard memorial lecture. J. Child. Psychol. Psychiatry.

[23] Pueschel S., Hopmann M., Kaiser A., Gray D. (1993). Speech and language abilities of children with down syndrome. Enhancing Children’s Communication: Research Foundations for Interventions.

[24] Turner S., Alborz A. (2003). Academic attainments of children with down’s syndrome: A longitudinal study. Br. J. Educ. Psychol..

[25] Carr J. (2005). Stability and change in cognitive ability over the life span: A comparison of populations with and without down’s syndrome. J. Intellect. Disabil. Res..

[26] Nadel L. (2003). Down’s syndrome: A genetic disorder in biobehavioral perspective. Genes Brain Behav..

[27] Abbeduto L., Warren S.F., Conners F.A. (2007). Language development in down syndrome: From the prelinguistic period to the acquisition of literacy. Ment. Retard. Dev. Disabil. Res. Rev..

[28] Chapman R.S. (2006). Language learning in down syndrome: The speech and language profile compared to adolescents with cognitive impairment of unknown origin. Downs Syndr. Res. Pract..

[29] Chapman R.S., Hesketh L.J. (2000). Behavioral phenotype of individuals with down syndrome. Ment. Retard. Dev. Disabil. Res. Rev..

[30] Amaral D., Leavenex P., Andersen P., Morris R., Amaral D., Bliss T., O’Keefe J. (2007). Hippocampal neuroanatomy. The Hippocampus Book.

[31] Cohen N.J., Ryan J., Hunt C., Romine L., Wszalek T., Nash C. (1999). Hippocampal system and declarative (relational) memory: Summarizing the data from functional neuroimaging studies. Hippocampus.

[32] Diana R.A., Yonelinas A.P., Ranganath C. (2007). Imaging recollection and familiarity in the medial temporal lobe: A three-component model. Trends Cogn. Sci..

[33] Bontempi B., Laurent-Demir C., Destrade C., Jaffard R. (1999). Time-dependent reorganization of brain circuitry underlying long-term memory storage. Nature.

[34] Frankland P.W., Bontempi B. (2005). The organization of recent and remote memories. Nat. Rev. Neurosci..

[35] Hammond R.S., Tull L.E., Stackman R.W. (2004). On the delay-dependent involvement of the hippocampus in object recognition memory. Neurobiol. Learn Mem..

[36] McGaugh J.L. (2000). Memory--a century of consolidation. Science.

[37] Mizuno K., Giese K.P. (2005). Hippocampus-dependent memory formation: Do memory type-specific mechanisms exist?. J. Pharmacol. Sci..

[38] Carlesimo G.A., Marotta L., Vicari S. (1997). Long-term memory in mental retardation: Evidence for a specific impairment in subjects with down’s syndrome. Neuropsychologia.

[39] Vicari S., Bellucci S., Carlesimo G.A. (2000). Implicit and explicit memory: A functional dissociation in persons with down syndrome. Neuropsychologia.

[40] Caltagirone C., Nocentini U., Vicari S. (1990). Cognitive functions in adult down’s syndrome. Int. J. Neurosci..

[41] Devenny D.A., Hill A.L., Patxot O., Silverman W.P., Wisniewski K.E. (1992). Ageing in higher functioning adults with down’s syndrome: An interim report in a longitudinal study. J. Intellect. Disabil. Res..

[42] Ellis N.R., Woodley-Zanthos P., Dulaney C.L. (1989). Memory for spatial location in children, adults, and mentally retarded persons. Am. J. Ment. Retard..

[43] Jernigan T.L., Bellugi U., Sowell E., Doherty S., Hesselink J.R. (1993). Cerebral morphologic distinctions between williams and down syndromes. Arch. Neurol..

[44] Mangan P.A. (1992). Spatial Memory Abilities and Abnormal Development of the Hippocampal Formation in down Syndrome. Doctoral Dissertation.

[45] Roberts L.V., Richmond J.L. (2015). Preschoolers with down syndrome do not yet show the learning and memory impairments seen in adults with down syndrome. Dev. Sci..

[46] Miyake A., Emerson M.J., Friedman N.P. (2000). Assessment of executive functions in clinical settings: Problems and recommendations. Semin. Speech Lang..

[47] Lanfranchi S., Carretti B., Spano G., Cornoldi C. (2009). A specific deficit in visuospatial simultaneous working memory in down syndrome. J. Intellect. Disabil. Res..

[48] Lanfranchi S., Cornoldi C., Vianello R. (2004). Verbal and visuospatial working memory deficits in children with down syndrome. Am. J. Ment. Retard..

[49] Lanfranchi S., Jerman O., Dal Pont E., Alberti A., Vianello R. (2010). Executive function in adolescents with down syndrome. J. Intellect. Disabil. Res..

[50] Rowe J., Lavender A., Turk V. (2006). Cognitive executive function in down’s syndrome. Br. J. Clin. Psychol..

[51] Kogan C.S., Boutet I., Cornish K., Graham G.E., Berry-Kravis E., Drouin A., Milgram N.W. (2009). A comparative neuropsychological test battery differentiates cognitive signatures of fragile x and down syndrome. J. Intellect. Disabil. Res..

[52] Nelson L.D., Scheibel K.E., Ringman J.M., Sayre J.W. (2007). An experimental approach to detecting dementia in down syndrome: A paradigm for alzheimer’s disease. Brain Cogn..

[53] Das J.P., Divis B., Alexander J., Parrila R.K., Naglieri J.A. (1995). Cognitive decline due to aging among persons with down syndrome. Res. Dev. Disabil..

[54] Baddeley A.D. (1986). Working Memory.

[55] Baddeley A.D., Baddeley A.D., Kopelman M.D., Wilson B.A. (2003). The psychology of memory. The Handbook of Memory Disorders.

[56] Lezak M.D., Howieson D.B., Bigler E.D., Tranel D. (2012). Neuropsychological Assessment.

[57] Chi M.T. (1977). Age differences in memory span. J. Exp. Child Psychol..

[58] Hulme C., Mackenzie S. (1992). Working Memory and Severe Learning Disabilities.

[59] Jarrold C., Baddeley A.D. (1997). Short-term memory for verbal and visuospatial information in down’s syndrome. Cogn. Neuropsychiatry.

[60] Jarrold C., Baddeley A.D., Phillips C.E. (2002). Verbal short-term memory in down syndrome: A problem of memory, audition, or speech?. J. Speech Lang. Hear Res..

[61] Mackenzie S., Hulme C. (1987). Memory span development in down’s syndrome, severely subnormal and normal subjects. Cogn. Neuropsychol..

[62] Marcell M.M., Harvey C.F., Cothran L.P. (1988). An attempt to improve auditory short-term memory in down’s syndrome individuals through reducing distractions. Res. Dev. Disabil..

[63] Marcell M.M., Weeks S.L. (1988). Short-term memory difficulties and down’s syndrome. J. Ment. Defic. Res..

[64] Numminen H., Service E., Ahonen T., Ruoppila I. (2001). Working memory and everyday cognition in adults with down’s syndrome. J. Intellect. Disabil. Res..

[65] Bird E.K., Chapman R.S. (1994). Sequential recall in individuals with down syndrome. J. Speech Hear Res..

[66] Jarrold C., Baddeley A.D., Hewes A.K. (1999). Genetically dissociated components of working memory: Evidence from down’s and williams syndrome. Neuropsychologia.

[67] Laws G. (2002). Working memory in children and adolescents with down syndrome: Evidence from a colour memory experiment. J. Child Psychol. Psychiatry.

[68] Wang P.P., Bellugi U. (1994). Evidence from two genetic syndromes for a dissociation between verbal and visual-spatial short-term memory. J. Clin. Exp. Neuropsychol..

[69] Silverstein A.B., Legutki G., Friedman S.L., Takayama D.L. (1982). Performance of down syndrome individuals on the stanford-binet intelligence scale. Am. J. Ment. Defic..

[70] Edgin J.O. (2013). Cognition in down syndrome: A developmental cognitive neuroscience perspective. Wiley Interdiscip. Rev. Cogn. Sci..

[71] Mosse E.K., Jarrold C. (2011). Evidence for preserved novel word learning in down syndrome suggests multiple routes to vocabulary acquisition. J. Speech Lang. Hear Res..

[72] Laws G., Briscoe J., Ang S.Y., Brown H., Hermena E., Kapikian A. (2015). Receptive vocabulary and semantic knowledge in children with sli and children with down syndrome. Child Neuropsychol..

[73] Cleave P., Bird E.K., Czutrin R., Smith L. (2012). A longitudinal study of narrative development in children and adolescents with down syndrome. Intellect. Dev. Disabil..

[74] Liogier d’Ardhuy X., Edgin J.O., Bouis C., de Sola S., Goeldner C., Kishnani P., Noldeke J., Rice S., Sacco S., Squassante L. (2015). Assessment of cognitive scales to examine memory, executive function and language in individuals with down syndrome: Implications of a 6-month observational study. Front. Behav. Neurosci..

[75] Esbensen A.J., Hooper S.R., Fidler D., Hartley S.L., Edgin J., d’Ardhuy X.L., Capone G., Conners F.A., Mervis C.B., Abbeduto L. (2017). Outcome measures for clinical trials in down syndrome. Am. J. Intellect. Dev. Disabil..

[76] Sattler A. (2001). Assessment of Children: Cognitive Applications.

[77] Bishop D.V.M. (1983). The Test for Reception of Grammar (trog).

[78] Bishop D.V.M. (2003). Test for Reception of Grammar: Trog-2.

[79] Bishop D.V.M. (2006). The children’s communication checklist. ccc–2 Manual.

[80] Wiig E.H., Secord W.A., Semel E. (1992). Clinical Evaluation of Language Fundamentals (Celf–Preschool).

[81] Semel E., Wiig E.H., Secord W.A. (1995). Clinical Evaluation of Language Fundamentals—Third Edition (celf–3).

[82] Korkman M., Kirk U., Kemp S. (1998). Nepsy: A Developmental Neuropsychological Assessment.

[83] Fernandez G., Weyerts H., Tendolkar I., Smid H.G., Scholz M., Heinze H.J. (1998). Event-related potentials of verbal encoding into episodic memory: Dissociation between the effects of subsequent memory performance and distinctiveness. Psychophysiology.

[84] Hermann B.P., Seidenberg M., Wyler A., Davies K., Christeson J., Moran M., Stroup E. (1996). The effects of human hippocampal resection on the serial position curve. Cortex.

[85] Kohler S., Black S.E., Sinden M., Szekely C., Kidron D., Parker J.L., Foster J.K., Moscovitch M., Winocour G., Szalai J.P. (1998). Memory impairments associated with hippocampal versus parahippocampal-gyrus atrophy: An mr volumetry study in alzheimer’s disease. Neuropsychologia.

[86] Thomas K.G., Hsu M., Laurance H.E., Nadel L., Jacobs W.J. (2001). Place learning in virtual space. Iii: Investigation of spatial navigation training procedures and their application to fmri and clinical neuropsychology. Behav. Res. Methods Instrum. Comput..

[87] De Sola S., de la Torre R., Sanchez-Benavides G., Benejam B., Cuenca-Royo A., Del Hoyo L., Rodriguez J., Catuara-Solarz S., Sanchez-Gutierrez J., Duenas-Espin I. (2015). A new cognitive evaluation battery for down syndrome and its relevance for clinical trials. Front. Psychol..

[88] Edgin J.O., Mason G.M., Allman M.J., Capone G.T., Deleon I., Maslen C., Reeves R.H., Sherman S.L., Nadel L. (2010). Development and validation of the arizona cognitive test battery for down syndrome. J. Neurodev. Disord..

[89] Rowe J.B., Owen A.M., Johnsrude I.S., Passingham R.E. (2001). Imaging the mental components of a planning task. Neuropsychologia.

[90] Kemp S.L., Kirk U., Korkman M. (2001). Essentials of Nepsy Assessment.

[91] Logan G.D., Cowan W.B., Davis K.A. (1984). On the ability to inhibit simple and choice reaction time responses: A model and a method. J. Exp. Psychol. Hum. Percept. Perform.

[92] Logan G.D., Schachar R., Tannock R. (1997). Impulsivity and inhibitory control. Psychol. Sci..

[93] Petrides M., Milner B. (1982). Deficits on subject-ordered tasks after frontal- and temporal-lobe lesions in man. Neuropsychologia.

[94] Case R., Kurland D.M., Goldberg J. (1982). Operational efficiency and the growth of short-term memory span. J. Exp. Child Psychol..

[95] Edgin J.O., Spano G., Kawa K., Nadel L. (2014). Remembering things without context: Development matters. Child Dev..

[96] Lee N.R., Fidler D.J., Blakeley-Smith A., Daunhauer L., Robinson C., Hepburn S.L. (2011). Caregiver report of executive functioning in a population-based sample of young children with down syndrome. Am. J. Intellect. Dev. Disabil..

[97] Edgin J.O., Anand P., Rosser T., Pierpont E.I., Figueroa C., Hamilton D., Huddleston L., Mason G., Spano G., Toole L. (2017). The arizona cognitive test battery for down syndrome: Test-retest reliability and practice effects. Am. J. Intellect. Dev. Disabil..

[98] Strauss E., Sherman E.M.S., Spreen O. (2006). A Compendium of Neuropsychological Tests: Administration, Norms and Commentary.

[99] Davidson M.C., Amso D., Anderson L.C., Diamond A. (2006). Development of cognitive control and executive functions from 4 to 13 years: Evidence from manipulations of memory, inhibition, and task switching. Neuropsychologia.

[100] Frith U., Frith C.D. (1974). Specific motor disabilities in down’s syndrome. J. Child Psychol. Psychiatry.

[101] Anand P., Sherman S., Reeves R.H., Rosser T., Capone G., Spano G., Edgin J.O. Assessing cognitive variability in down syndrome—Test-retest reliability & practice effects of the arizona cognitive test battery. Paper Presented at the DSMIG-USA Annual Symposium.

[102] Desmond J.E., Gabrieli J.D., Wagner A.D., Ginier B.L., Glover G.H. (1997). Lobular patterns of cerebellar activation in verbal working-memory and finger-tapping tasks as revealed by functional mri. J. Neurosci..

[103] Kaufman A.S., Kaufman N.L. (2004). Kaufmann Brief Intelligence Test.

[104] Edgin J.O., Pennington B.F., Mervis C.B. (2010). Neuropsychological components of intellectual disability: The contributions of immediate, working, and associative memory. J. Intellect. Disabil. Res..

[105] Bruininks R.H., Woodcock R.W., Weatherman R.F., Hill B.K. (1996). Scales of Independent Behavior-Revised (sib-r).

[106] Gioia G.A., Isquith P.K., Guy S.C., Kenworthy L. (2000). Test review behavior rating inventory of executive function. Child Neuropsychol..

[107] Aman M.G., Tasse M.J., Rojahn J., Hammer D. (1996). The nisonger cbrf: A child behavior rating form for children with developmental disabilities. Res. Dev. Disabil..

[108] Heller J.H., Spiridigliozzi G.A., Crissman B.G., McKillop J.A., Yamamoto H., Kishnani P.S. (2010). Safety and efficacy of rivastigmine in adolescents with down syndrome: Long-term follow-up. J. Child Adolesc. Psychopharmacol..

[109] Randolph C., Tierney M.C., Mohr E., Chase T.N. (1998). The repeatable battery for the assessment of neuropsychological status (rbans): Preliminary clinical validity. J. Clin. Exp. Neuropsychol..

[110] Bedard A.C., Martinussen R., Ickowicz A., Tannock R. (2004). Methylphenidate improves visual-spatial memory in children with attention-deficit/hyperactivity disorder. J. Am. Acad. Child Adolesc. Psychiatry.

[111] Semel E., Wiig E.H., Secord W.A. (2004). Clinical Evaluation of Language Fundamentals^®^-Preschool-2 (celf^®^-Preschool-2).

[112] Roid G.H., Miller L.J. (1997). Leiter International Performance Scale-Revised: Examiner’s Manual.

[113] Robbins T.W., James M., Owen A.M., Sahakian B.J., Lawrence A.D., McInnes L., Rabbitt P.M. (1998). A study of performance on tests from the cantab battery sensitive to frontal lobe dysfunction in a large sample of normal volunteers: Implications for theories of executive functioning and cognitive aging. Cambridge neuropsychological test automated battery. J. Int. Neuropsychol. Soc..

[114] Costa A.C., Scott-McKean J.J. (2013). Prospects for improving brain function in individuals with down syndrome. CNS Drugs.

[115] Costa A.C. (2011). On the promise of pharmacotherapies targeted at cognitive and neurodegenerative components of down syndrome. Dev. Neurosci..

[116] Costa A.C., Scott-McKean J.J., Stasko M.R. (2008). Acute injections of the nmda receptor antagonist memantine rescue performance deficits of the ts65dn mouse model of down syndrome on a fear conditioning test. Neuropsychopharmacology.

[117] Lockrow J., Boger H., Bimonte-Nelson H., Granholm A.C. (2011). Effects of long-term memantine on memory and neuropathology in ts65dn mice, a model for down syndrome. Behav. Brain Res..

[118] Rueda N., Llorens-Martin M., Florez J., Valdizan E., Banerjee P., Trejo J.L., Martinez-Cue C. (2010). Memantine normalizes several phenotypic features in the ts65dn mouse model of down syndrome. J. Alzheimers Dis..

[119] Scott-McKean J.J., Roque A.L., Surewicz K., Johnson M.W., Surewicz W.K., Costa A.C.S. (2018). Pharmacological modulation of three modalities of ca1 hippocampal long-term potentiation in the ts65dn mouse model of down syndrome. Neural Plast..

[120] Scott-McKean J.J., Costa A.C. (2011). Exaggerated nmda mediated ltd in a mouse model of down syndrome and pharmacological rescuing by memantine. Learn Mem..

[121] Woods S.P., Delis D.C., Scott J.C., Kramer J.H., Holdnack J.A. (2006). The california verbal learning test--second edition: Test-retest reliability, practice effects, and reliable change indices for the standard and alternate forms. Arch. Clin. Neuropsychol..

[122] Sample Size Calculator. https://www.ai-therapy.com/psychology-statistics/sample-size-calculator.

[123] Hanney M., Prasher V., Williams N., Jones E.L., Aarsland D., Corbett A., Lawrence D., Yu L.M., Tyrer S., Francis P.T. (2012). Memantine for dementia in adults older than 40 years with down’s syndrome (meadows): A randomised, double-blind, placebo-controlled trial. Lancet.

[124] Spiridigliozzi G.A., Hart S.J., Heller J.H., Schneider H.E., Baker J.A., Weadon C., Capone G.T., Kishnani P.S. (2016). Safety and efficacy of rivastigmine in children with down syndrome: A double blind placebo controlled trial. Am. J. Med. Genet. A.

[125] Kishnani P.S., Heller J.H., Spiridigliozzi G.A., Lott I., Escobar L., Richardson S., Zhang R., McRae T. (2010). Donepezil for treatment of cognitive dysfunction in children with down syndrome aged 10–17. Am. J. Med. Genet. A.

[126] Fidler D.J., Nadel L. (2007). Education and children with down syndrome: Neuroscience, development, and intervention. Ment. Retard. Dev. Disabil. Res. Rev..

[127] Silverman W. (2007). Down syndrome: Cognitive phenotype. Ment. Retard. Dev. Disabil. Res. Rev..

